# The Effects of Korea Red Ginseng on Inflammatory Cytokines and Apoptosis in Rat Model with Chronic Nonbacterial Prostatitis

**DOI:** 10.1155/2019/2462561

**Published:** 2019-01-14

**Authors:** Sang Wook Kang, Je-Hoon Park, Hosik Seok, Hae Jeong Park, Joo-Ho Chung, Chang-Ju Kim, Young Ock Kim, Young Rok Han, DongWhan Hong, Young Sik Kim, Su Kang Kim

**Affiliations:** ^1^Kohwang Medical Research Institute, School of Medicine, Kyung Hee University, Seoul, Republic of Korea; ^2^Department of Surgery, International St. Mary's Hospital, College of Medicine, Catholic Kwandong University, Incheon, Republic of Korea; ^3^Department of Physiology, College of Medicine, Kyung Hee University, Seoul, Republic of Korea; ^4^Development of Bio-Environmental Chemistry, College of Agriculture and Life Sciences, Chungnam National University, Daejeon, Republic of Korea; ^5^Department of Physical Medicine & Rehabilitation, Graduate School, Kyung Hee University, Seoul, Republic of Korea; ^6^Management Research Institute, Kyung Hee University, Seoul, Republic of Korea; ^7^Department of Biomedical Laboratory Science, Catholic Kwandong University, Gangneung, Republic of Korea

## Abstract

Chronic prostatitis typically occurs in aging men, and its symptoms include frequent and painful urination. In recent study, several studies have shown that Korean red ginseng (KRG) can be used in the prevention and treatment of various diseases. The objective of this study is to investigate whether KRG can play a role in repressing the development of chronic nonbacterial prostatitis (CNP) in male Wistar rats. To induce CNP, rats were castrated and beta-estradiol (0.25 mg/kg) was subcutaneously (s.c.) injected daily. 7-week-old male Wistar rats were divided into 5 groups (the normal group, CNP group, positive group, and KRG group (0.25g/kg) and another KRG (0.50g/kg) group. After 4 weeks, all rats were sacrificed and their prostate and serum were analyzed. Compared to the positive group, the KRG groups (0.25g/kg and 0.50g/kg) showed similar protective properties on CNP based on the histopathologic morphology of the prostate and the inflammation cytokines in the prostate tissue. Also, results of the immunohistochemistry staining showed that expression levels of vascular endothelial growth factor A (VEGFA), interleukin 6 (IL6), interleukin 1 beta (IL-1ß), tumor necrosis factor (TNF-alpha), and cytochrome c oxidase subunit II (COX2) were also decreased in KRG group (0.25g/kg) and KRG group (0.50g/kg). These results suggested that KRG inhibited the development of CNP and might a useful herbal treatment or functional food for CNP.

## 1. Introduction

Chronic prostatitis is a common disease in aged men [1]. The disease causes a diverse range and degree of symptoms, including voiding irritation, pelvic region discomfort, and sexual dysfunctions [2–4].

Chronic prostatitis is classified as acute, chronic, bacterial, and nonbacterial prostatitis. More than 90% of all prostatitis patients are affected by chronic nonbacterial prostatitis (CNP) [5]. Despite the high incidence of CNP, the cause of the disease has not been verified, and it is known to be difficult to treat. It is a morbid illness that causes mental dissatisfaction and negatively impacts an individual's daily quality of life.

The disease is characterized by chronic pelvic pain and possibly voiding symptoms with no evidence of urinary tract infection [4]. Although it is not an infection, it may cause pathologic changes including dysmorphic prostatic glandular ducts, gland atrophy, interstitial fibrosis, and infiltrations of polymorphonuclear neutrophils, lymphocytes, and monocytes. CNP is an inflammatory disease in which inflammatory cells are found in microscopic examinations of the prostate gland with our any detection of a causative organism [1].

Inflammation is a biologic defense reaction against a stimulant substance. Various cytokines are involved in inflammatory reactions. Expressions of proinflammatory cytokine such as tumor necrosis factor-*α* (TNF-*α*), vascular endothelial growth factor A (VEGFA), interleukin 6 (IL6), and interleukin 1 beta (IL-1ß) are induced in the macrophage inflammatory response by external stimulation. Nitric oxide (NO) is produced by stimulating the expression of inducible nitric oxide synthase (iNOS) and cyclooxygenase-2 (COX-2) [6].

Korea red ginseng (KRG) has been used safely in human for a long time. KRG has been traditionally used in Korea and East Asian countries for treatment of diverse diseases [7]. It is a heat-modified product of the Ginseng Radix (*Panax ginseng*) root. KRG was originally developed for the sole purpose of improving the long-term preservation of the root, but it was found to contain the active elements of ginseng saponins or ginsenosides, of which the original root does not [8]. These elements are known to increase the efficacy of ginseng. The KRG known so far has been reported to be effective as an antioxidant, antibacterial agent, anti-inflammatory action agent, blood circulation enhancer, cancer preventing agent, and infectious defense and immunity enhance [9–13]. KRG is also commonly used for male rejuvenation [14, 15] and, to treat urinary discomfort in the elderly, as it is known in Korea as a herb that improves vigor and stamina [16, 17].

The aim of the present study is to investigate the effects of orally administrated KRG in CNP rat model based on the histopathologic morphology of the prostate and inflammation cytokines in the prostate tissue.

## 2. Methods

### 2.1. Preparation of the KRG

The material of KRG was provided from Korea Ginseng Corp (KGC, Korea).

### 2.2. Animals

The animals used in this study were 7-week-old male Wistar rats (RaonBio Inc., Korea) with an average body weight of 250±10g. The animal room was maintained at 22±2°C with 40-70% relative humidity. The room lighting consisted of 12h/12h of light and dark. All experiments were carried out according to the protocols approved by the Animal Care Committee of the Animal Center at Kyung Hee University in accordance with guidelines from the Korean National Health Institute of Health Animal Facility (KHUASP-16-019).

### 2.3. Induction of CNP and Treatments

After the orchiectomy, rats that were injected with beta estradiol (0.25 mg/kg, Sigma-Aldrich Co., St. Louis, MO, USA) (20 mg/kg) for 4 weeks were shown to have prostatitis. The rats were then divided into five groups (*n* = 5): (A) a normal control group; (B) a CNP group: CNP that induced 17B-estradiol group through subcutaneous injection; (C) a positive group: CNP that induced 17B-estradiol group through subcutaneous injection + testosterone, injected subcutaneously; (D) a KRG (0.25g/kg) group: CNP that induced 17B-estradiol group through subcutaneous injection + KRG (0.25g/kg); (E) and another KRG (0.50g/kg) group: CNP that induced 17B-estradiol group through subcutaneous injection + KRG (0.50g/kg), administered orally. The experiment was performed with the drug administration and subcutaneous injection for 4 weeks. After 4 weeks, drug administration and subcutaneous injection were stopped.

### 2.4. Blood Collection and Biochemical Analysis

To collect blood, rats were anesthetized and blood was drawn from their hearts into the serum separate tube (SST) tubes. The serum was separated from the blood by centrifuge at 3,000 rpm. Serum was gathered immediately and stored at -70°C. The level of glutamic oxaloacetic transaminase (GOT), glutamic pyruvic transaminase (GPT), blood urea nitrogen (BUN), creatinine, and *γ*-GTP in serum were tested by Greenlab (Seoul, Korea).

### 2.5. Tunnel Assay in Prostate Tissue

The prostate tissues in each group were dewaxed with xylenes and rehydrated through an ethanol series. Tunnel reactions were performed with the In Situ Cell Death Detection POD Kit (Roche USA) according to the manufacturer's protocol. The prostate tissues after the Tunnel reactions were dehydrated, mounted, and examined under a microscope.

### 2.6. Nitric Oxide (NO) Assay in Serum

NO accumulation was used as an indicator of NO production. Its level in serum was determined using the Griess reagent (Promega, USA, Griess Reagent system). Briefly, serum (50*μ*l) was mixed with the same volume of Griess reagent (1% sulfanilamide and 0.1% N-(1-naphthyl)-ethylenediamine dihydrochloride in 5% phosphoric acid) for 10 min, and absorbance was measured at 520 nm.

### 2.7. RNA Extraction and Reverse Transcriptase-Polymerase Chain Reaction (RT-PCR)

Total RNA was isolated from the prostate tissues of each mouse using Trizol (Invitrogen, CA, USA), according to the manufacturer's instructions. An aliquot of total RNA was reverse transcribed using MMuLV reverse transcriptase and Taq DNA polymerase (Promega, Madison, WI, USA), respectively. Primers for VEGFA, IL6, IL-1ß, TNF-*α*, and COX2 were designed in reverse transcription (RT- PCR) according to previous studies [18, 19].

### 2.8. Immunohistochemistry (IHC)

Immunohistochemistry (IHC) staining was performed for prostate tissue section at thickness of 10*μ*m. First, paraffin was removed from slides through a deparaffinization process. To avoid endogenous peroxidase activity, slides were incubated with 3% of H_2_O_2_ solution in methanol at room temperature for 15 minutes and then washed with PBS for 3 times (5 minutes each). Slides were prerestrained with normal goat or rabbit serum for 1hr. In primary antibody reaction stage, slides were incubated carefully with anti-VEGFA, anti-TNF-alpha, anti-IL1*β*, anti-IL-6, and anti-COX2 (Santa Cruz Biotechnology Inc., Santa Cruz, CA, USA) in a 1:500 dilution for a night at 4°C and washed with PBS for 3 times, 5 minutes each. For second antibody reaction, the slides were incubated with biotinylated secondary antibodies (1:1000) at room temperature for 1h and washed with PBS three times, five minutes each. Finally, diaminobenzidine tetrahydrochloride substrate (DAB) staining with streptavidin-HRP was performed to visualize antigen-antibody reaction. For each section, three different random fields were examined at least ×100 magnification.

### 2.9. Statistical Analyses

All values were presented as the mean±standard error (SE). Significant differences among the groups were statistically analyzed by using the one-way analysis of variances (ANOVA), followed by a nonparametric post-Tukey test. All* p* values were two-tailed. A p value of less than 0.05 was considered statistically significant. All statistical analyses were performed using SPSS 22.0 for Windows.

## 3. Results

### 3.1. Effect of KRG on Weight Change

**Figure 1:**
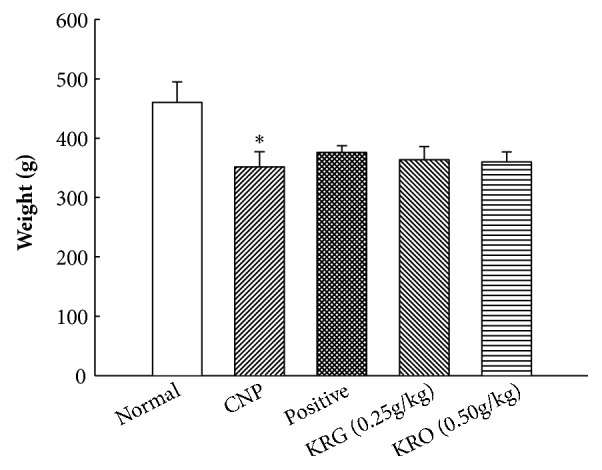
**Effects of Korea red ginseng (KRG) on body weight in each group**. Normal, normal group; CNP, chronic nonbacterial prostatitis group; Positive, estradiol induced CNP with testosterone; KRG (0.25g/kg), beta-estradiol induced CNP with KRG (0.25g/kg); KRG (0.5g/kg), estradiol induced CNP with KRG (0.5g/kg). *∗* P < 0.05, compared with the normal group and # P < 0.05, compared with the CNP group.

The changes in body weight are shown in Figure 1. Final body weight was measured at 30 days after treatment. The final body weights in each group (CNP, positive, and KRG groups) had decreased compared to that in the normal group. However, differences among CNP, positive, and KRG groups were not significant (p>0.05).

### 3.2. Effect of KRG on Levels of GOT, GPT, BUN, Creatinine, and *γ*-GTP in Serum

Serum levels of GOT, GPT, BUN, creatinine, and *γ*-GTP are shown in Table 1. Levels of GOT in the normal, CNP, positive, KRG (0.25g/kg), and KRG (0.50g/kg) groups were 77.0±9.2, 75.6±3.3, 76.0±10.3,100.1±13.1, and 91.1±13.7 IU/L, respectively. Levels of GPT in the normal, CNP, positive, KRG (0.25g/kg), and KRG (0.50g/kg) groups were 28.0±10.1, 41.5±9.6, 26.2±4.9, 34.7±16.8, and 38.6±20.1 IU/L, respectively. Levels of BUN in the normal, CNP, positive, KRG (0.25g/kg), and KRG (0.5g/kg) groups were 18.0±1.0, 19.3±2.5, 16.7±1.5, 22.0±1.0, and 16.7±3.2 mg/dl, respectively. Levels of creatinine in the normal, CNP, positive, KRG (0.25g/kg), and KRG (0.5g/kg) groups were 0.49±0.07, 0.55±0.04, 0.59±0.02, 0.50±0.04, and 0.48±0.07 mg/dl, respectively. And levels of *γ*-GTP were less than 3 IU/L in all groups. Differences among groups were not statistically significant (p>0.05). These results indicated that KRG did not have toxicity in the animal model.

### 3.3. Effects of KRG on Apoptosis in Prostate Tissue Using Tunnel Assay

**Figure 2:**
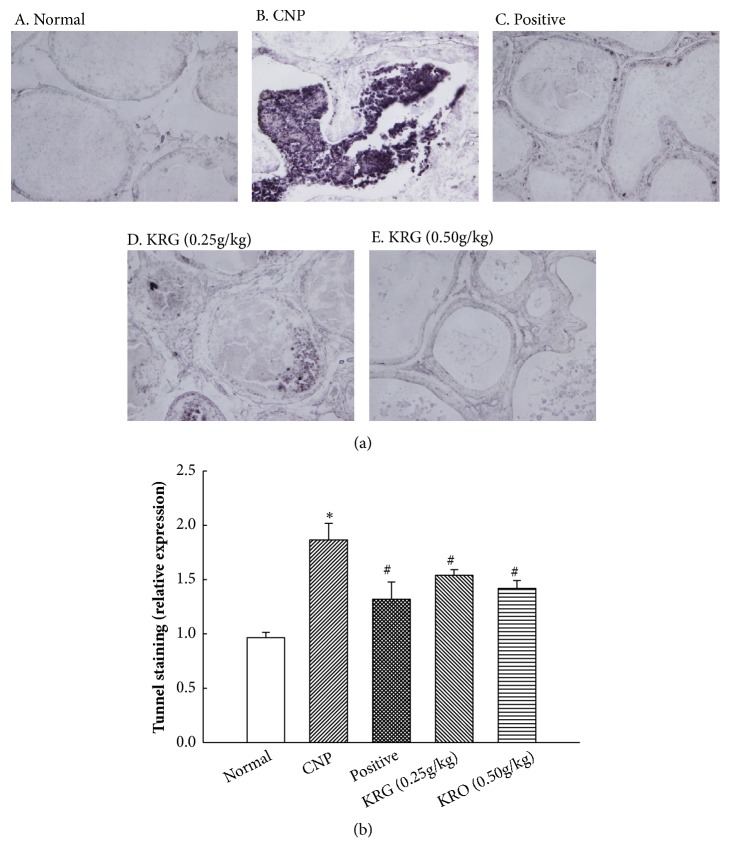
**Tunnel staining in prostate tissue in each group (x100)**. Normal, normal group; CNP, chronic nonbacterial prostatitis group; Positive, estradiol induced CNP with testosterone; KRG (0.25g/kg), beta-estradiol induced CNP with KRG (0.25g/kg); KRG (0.5g/kg), estradiol induced CNP with KRG (0.5g/kg). *∗* P < 0.05, compared with the normal group and # P < 0.05, compared with the CNP group.

As shown in Figures 2(a) and 2(b), apoptosis of the prostate tissue among the groups was examined. The apoptosis of prostate tissue had significantly increased in the CNP group. The apoptosis of prostate tissue in the positive and KRG (0.25g/kg and 0.50g/kg) groups had significantly decreased compared to that in the CNP group (p<0.05).

### 3.4. Effects of KRG on Nitric Oxide Level in Serum

**Figure 3:**
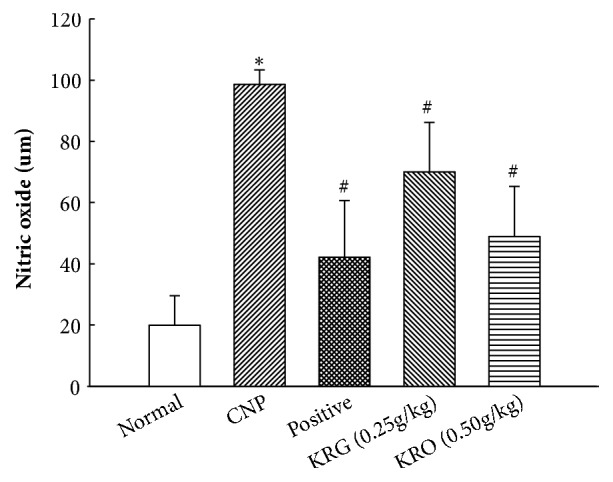
**Analysis of nitric oxide (NO) in serum in each group**. Normal, normal group; CNP, chronic nonbacterial prostatitis group; Positive, estradiol induced CNP with testosterone; KRG (0.25g/kg), beta-estradiol induced CNP with KRG (0.25g/kg); KRG (0.5g/kg), estradiol induced CNP with KRG (0.5g/kg). *∗* P < 0.05, compared with the normal group and # P < 0.05, compared with the CNP group.

Serum levels of NO in the normal, CNP, positive, KRG (0.25g/kg), and KRG (0.50g/kg) groups were 19.89±9.7, 98.6±4.7, 42.2±18.5, 70.0±16.1, and 48.9±16.4 uM (Figure 3). The level of NO in the CNP group was significantly higher compared to that in the normal group (p<0.05). And the levels of NO in the positive and KRG groups (0.25 g/kg and 0.5 g/kg) were significantly lower than those in the CNP group (*p* < 0.05).

### 3.5. Effect of KRG on mRNA Expression Levels of Inflammation Cytokines in Prostate Tissue Using RT-PCR

**Figure 4:**
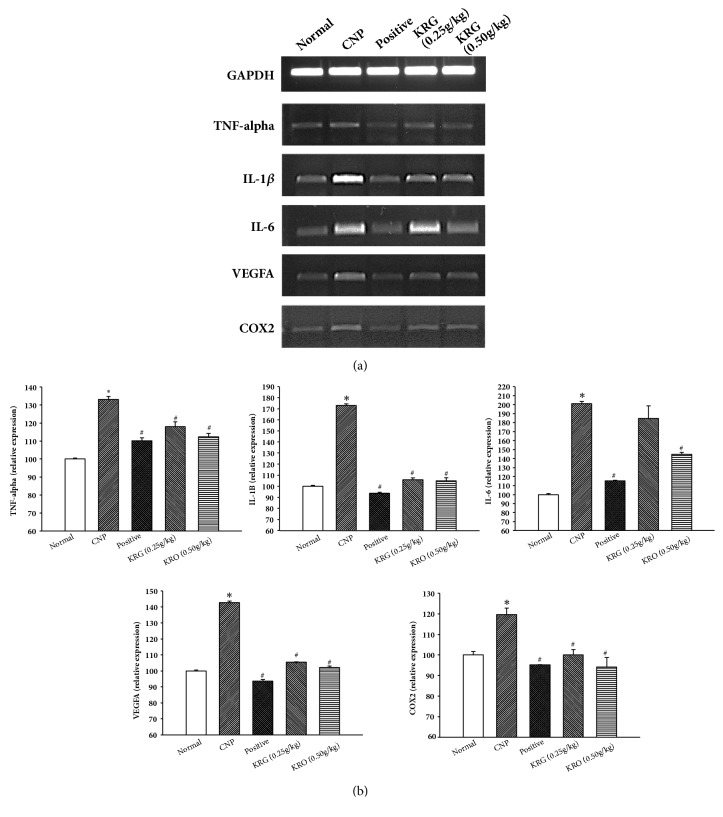
**The expressions of TNF-alpha, IL-1** **β** **, IL-6, VEGFA, and COX2 by RT-PCR in each group**. Normal, normal group; CNP, chronic nonbacterial prostatitis group; Positive, estradiol induced CNP with testosterone; KRG (0.25g/kg), beta-estradiol induced CNP with KRG (0.25g/kg); KRG (0.5g/kg), estradiol induced CNP with KRG (0.5g/kg). *∗* P < 0.05, compared with the normal group and # P < 0.05, compared with the CNP group.

The measurements of mRNA expression levels of VEGFA, TNF-alpha, IL-1*β*, IL-6, and COX2 were performed using RT-PCR. The mRNAs of VEGFA, TNF-alpha, IL-1*β*, IL-6, and COX2 were amplified by RT-PCR. As shown in Figure 4, mRNA expression levels of VEGFA, TNF-alpha, IL1*β*, IL-6, and COX2 in the CNP group at 4 weeks were higher than those of the normal group (Figure 4(a)). In the positive and KRG (0.25g/kg and 0.50g/kg) groups at 4 weeks, mRNA expression levels of VEGFA, TNF-alpha, IL1*β*, IL-6, and COX2 had significantly decreased compared to those in the CNP group (p<0.05) (Figure 4(b)).

### 3.6. Effect of KRG on Expression of Inflammation Cytokines in Prostate Tissue Using IHC

**Figure 5:**
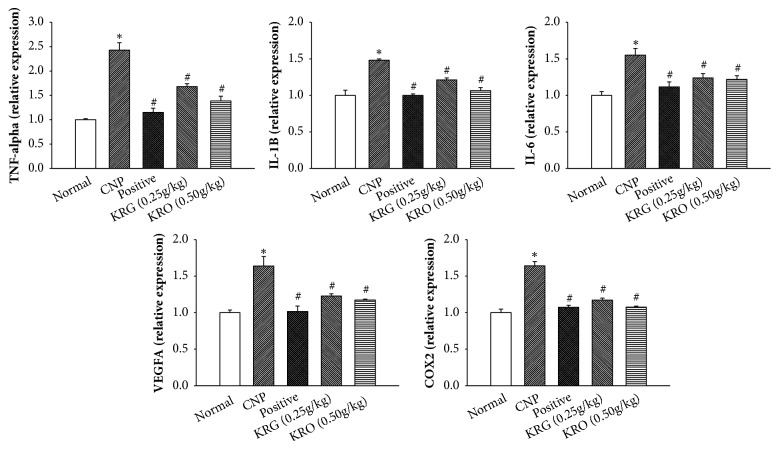
**The expressions of TNF-alpha, IL-1** **β** **, IL-6, VEGFA, and COX2 by immunohistochemistry in each group**. Normal, normal group; CNP, chronic nonbacterial prostatitis group; Positive, estradiol induced CNP with testosterone; KRG (0.25g/kg), beta-estradiol induced CNP with KRG (0.25g/kg); KRG (0.5g/kg), estradiol induced CNP with KRG (0.5g/kg). *∗* P < 0.05, compared with the normal group, and # P < 0.05, compared with the CNP group.

To examine the effects of KRG on expression of inflammation cytokines (VEGFA, TNF-alpha, IL-1*β*, IL-6, and COX2) in prostate tissue, IHC was performed. As shown in Figure 5, expression levels of inflammation cytokines (VEGFA, TNF-alpha, IL-1*β*, IL-6, and COX2) in the CNP group were higher than those in the normal group (p<0.05). Proteins expression levels of VEGFA, TNF-alpha, IL-1*β*, IL-6, and COX2 in both positive and KRG (0.25g/kg and 0.50g/kg) groups had decreased compared to those in the CNP group (p<0.05).

## 4. Discussion

In this study, we examined the effect of KRG on CNP using the E2-induced CNP animal model. Our results showed the effects of KRG on the inflammation and morphology of prostate tissue in case of the CNP.

Weight loss was the first difference observed when comparing the normal group against the other groups. Weight difference between the normal and E2 induced prostatitis groups, however, has already been reported in previous studies. These studies suggest that E2 may be causative or that prostatitis itself may play a role in such reduction in body weight [20, 21]. In our study, positive and the KRG groups also showed weight loss, which is E2 or prostatitis.

To examine the effect of KRG on hepatotoxicity, the serum levels of GOT, GPT, BUN, creatinine, and *γ*-GTP were measured. Most of the serum levels in the CNP, positive, and the KRG groups were not significantly different from those in the normal group. The only GOT level in of the KRG (0.25g/kg) group was statistically higher than that in the other groups but it was within the normal range [22]. These results suggest that KRG has no toxicity in the CNP animal model.

The results of TUNEL staining show clear differences among the groups. The infiltration of inflammatory cells, destruction of prostate gland tissue, and a lot of apoptotic cells were observed in the CNP group. In the KRG group, it was observed that these changes of prostate tissue were significantly reduced. At higher concentrations of KRG, tissue changes and apoptotic cells were hardly observed. In the relative expression analysis, evidence of apoptosis had significantly increased in the CNP group and decreased in the positive and KRG groups compared with that in the CNP group. This result suggests that KRG has an antiapoptotic and anti-inflammatory effects on CNP.

To investigate the anti-inflammatory effect of KRG, levels of NO and proinflammatory cytokines were analyzed. NO is considered as a proinflammatory mediator [23]. The level of NO was significantly increased in the CNP group and decreased in the other groups compared with that in the CNP group. The results of RT-PCR and IHC for the proinflammatory cytokine showed a similar tendency. The mRNA and protein expression levels of TNF-alpha, IL-1*β*, IL-6, VEGFA, and COX2 in positive and the KRG groups were significantly lower than those in the CNP group. In addition, higher concentrations of KRG showed more effective anti-inflammatory responses in all cytokines (TNF-alpha, IL-1*β*, IL-6, VEGFA, and COX2).

Many previous studies have revealed that KRG has anti-inflammatory and antiapoptotic properties through various pathways. Red ginseng attenuated inflammatory responses through inhibition of the MAPKs/NF-*κ*B/c-Fos pathways in the lung tissue [24]. In atopic dermatitis animal model, inflammatory responses were reduced through the suppression of the p70 ribosomal protein S6 kinase pathway by red ginseng [25]. In a previous study of FK506-induced nephrotoxicity in LLC-PK1 cells, KRG was also shown to play a protective role. KRG was showed antiapoptotic properties via the inhibition of p38 phosphorylation and activation of caspase, as well as an anti-inflammatory property via the inhibition of TLR-4 expression. Our results are consistent with these previous studies [26]. We showed the antiapoptotic effect of KRG using TUNEL assay and the anti-inflammatory effect of KRG by confirming the reduction of inflammatory mediator NO and the decrease of proinflammatory cytokine using RT-PCR and IHC.

## 5. Conclusions

Our results suggest that KRG plays protective effects against CNP in rat model via the suppressing expression of TNF-alpha, IL-1*β*, IL6, VEGFA, and COX2. In conclusion, KRG could be a useful agent for the prevention or treatment of CNP.

## Figures and Tables

**Table 1:** Analysis of GOT, GPT, BUN, creatinine, and *γ*-GTP level in serum level.

Group	GOT	GPT	BUN	Creatinine	*γ*-GTP
	(IU/L)	(IU/L)	(mg/dl)	(mg/dl)	(U/L)
Normal	77.0±9.2	28.0±10.1	18.0±1.0	0.49±0.07	<3
CNP	75.6±3.3	41.5±9.6	19.3±2.5	0.55±0.04	<3
Positive	76.0±10.3	26.2±4.9	16.7±1.5	0.59±0.02	<3
KRG (0.25g/kg)	100.1±13.1#	34.7±16.8	22.0±1.0	0.50±0.04	<3
KRG (0.50g/kg)	91.1±13.7	38.6±20.1	16.7±3.2	0.48±0.07	<3

Data are presented as mean ± SE (*n*=6).

^#^P < 0.05, compared with the CNP group.

## Data Availability

The data used to support the findings of this study are included within the article.
